# Learning changes in educational animation: visuospatial working memory is more predictive than subjective task load

**DOI:** 10.3389/fpsyg.2024.1389604

**Published:** 2024-09-19

**Authors:** Rolf Ploetzner

**Affiliations:** Department of Psychology, University of Education Freiburg, Freiburg, Germany

**Keywords:** animation, learning, visual working memory, spatial working memory, task load

## Abstract

Current theories suggest that visual and spatial processes in working memory are crucial for learning from animation. However, despite over three decades of research on learning from animation, little is known about how visuospatial working memory relates to learning. Instead, animation research often relies on subjective task load to explain and predict learning performance. To better understand how visuospatial working memory and learning from animation are related, a within-subjects study was conducted. Eighty six students learned from two animations of different complexity. The students’ performance on visual learning tasks, visual and spatial working memory capacity, and perceived task load were assessed. Hierarchical regression analyses show that visuospatial working memory capacity is more critical for learning from a complex animation than for learning from a less complex animation. Moreover, visuospatial working memory capacity predicts learning from a complex animation significantly better than subjective task load. The effect size is large. The results provide a coherent picture of the relationships between learning task demands, learners’ visuospatial working memory, perceived task load and learning performance. They not only allow for a more accurate prediction of learning from animation but can also help to tailor the design and use of animations to the learners’ cognitive resources.

## 1 Introduction

Animation is widely used in education. For learning from animation to be successful, the design and use of animation should take into account the perceptual and cognitive resources of learners. Current theories consider perceptual processing to be an essential component of learning from animation. It involves the identification of events and relationships between events in the animated display. According to Lowe (1999), this requires the learner to perceptually extract information from the display at a given time, store it in memory until more information appears on the display, and then compare and contrast the memorized and external representations (see also Ploetzner and Lowe, 2017). According to Luck and Hollingworth (2008) and Schurgin (2018), an important function of visuospatial working memory is to bridge spatial and temporal gaps in our perception and to store features of visual targets for subsequent comparisons. Given this parallelism, it is reasonable to assume that visuospatial working memory plays a central role in learning from animation.

Current theories of multimedia learning such as the Cognitive Theory of Multimedia Learning (Mayer, 2021, 2022) and the Cognitive Load Theory (Paas and Sweller, 2022) recognize the role of working memory in learning. Commonly, these theories are based on Baddeley’s (1999) model of working memory, which consists of the central executive, the verbal working memory (called the phonological loop), the episodic buffer, and the visuospatial working memory (called the visuospatial sketchpad). Several researchers have suggested that visuospatial working memory should be further subdivided into a visual and a spatial subcomponent (e.g., Logie and Pearson, 1997; see also Baddeley et al., 2021).

While the role of working memory has received some attention in research on multimedia learning (for reviews see Gyselinck et al., 2008; Schüler et al., 2011), it has been largely neglected in research on learning from animation. Instead, animation research often relies on subjective measures of task load, such as cognitive load (Paas and Sweller, 2022), to explain and predict learning performance. A deeper understanding of the role of visuospatial working memory in learning from animation would help not only to tailor the design and use of animation to learners’ perceptual and cognitive resources, but also to validate and further develop theoretical models of learning from animation. Tailoring the design and use of animations to learners’ visuospatial working memory rather than their task load ratings has at least three advantages:

1. Visuospatial working memory is easier to assess objectively than task load. There are several standardized instruments for the objective assessment of visuospatial working memory capacity, such as the Visual Patterns Test (Della Sala et al., 1997) and the Corsi Block Tapping Test (Corsi, 1972). Although attempts have been made to objectively assess task load using neurophysiological and dual-task methods (for reviews see Brünken et al., 2003; Korbach et al., 2017), these methods are difficult to apply. Therefore, in most cases, task load is assessed using subjective ratings (for reviews see Krieglstein et al., 2022; Paas et al., 2003).
2. It is more economical to assess visuospatial working memory than task load. While visuospatial working memory remains stable across successive learning tasks, task load, by definition, varies from learning task to learning task. Therefore, assessments of visuospatial working memory can be used to tailor multiple learning tasks, whereas assessments of task load are specific to individual learning tasks.
3. Visuospatial working memory can be assessed at a more appropriate point in time than task load, namely prior to the administration of a learning task. The result of the assessment can then be used to tailor the learning task to the learner’s cognitive resources. Task load, in contrast, is assessed either while a learning task is being performed or after a learning task has been completed. This makes it difficult to use task load to adapt learning tasks.

Therefore, this study focuses on two main research questions. First, how does visuospatial working memory relate to learning performance when learning from animation? Second, how does visuospatial working memory predict learning performance compared to subjective task load?

## 2 Theoretical and empirical background

### 2.1 Learning from animation

According to Hegarty and Just (1993) and Hegarty et al. (2003), two mental models are constructed during learning from animation: a kinematic model and a conceptual model. A kinematic model makes up an analog mental representation of the displayed changes and thus represents the visuospatial and spatiotemporal organization of the animation. It unfolds in time and the sequence of events it represents corresponds to the sequence of displayed changes. A conceptual model consists of a symbolic mental representation of relationships, concepts, and principles that are relevant to the animated subject matter and that conceptually describe and explain the changes in the display.

In the Animation Processing Model (APM), Lowe and Boucheix (2008; see also Lowe et al., 2022) describe the stepwise construction of a kinematic model using three phases: (1) the identification of individual event units (i.e., individual graphic entities and their behavior), (2) the combination of event units into local relational structures, and (3) the construction of more extensive relational structures that eventually encompass the entire spatial and temporal extent of the animation. Two further phases are distinguished in the creation of a conceptual model: (4) the assignment of functional roles to the identified relational structures and (5) the elaboration of the function of the animated system under different operating conditions.

While the early phases of animation processing are largely based on bottom-up perceptual processes, the later phases are mainly based on top-down conceptual processes. According to Lowe and Boucheix (2008), the initial identification of event units forms the basis for subsequent processing and is likely to be challenging for learners due to competition for limited perceptual and cognitive resources. Thus, learners’ visuospatial working memory can be expected to directly influence the early phases of animation processing and to indirectly influence the later phases as well. But what is known about the role of visuospatial working memory in learning from animation?

In their review of research on the role of working memory in learning from text and pictures, Gyselinck et al. (2008) summarize that, overall, the studies analyzed indicate that visuospatial working memory is involved whenever visuospatial information must be processed. This can be the case when visual information is perceived, reconstructed from knowledge, or constructed through imagery. Although learning from animation is not a focus of this review, it is reasonable to assume that these findings also apply to learning from animation. In a further review, Schüler et al. (2011) conclude that both capacity-based methods and dual-task methods for assessing working memory confirm the assumption that different working memory subsystems serve different functions in multimedia learning. Especially, the studies reviewed confirm that visuospatial working memory is involved in processing static and dynamic visualizations such as animations.

More recently, Castro-Alonso et al. (2019) have described how visuospatial ability can influence learning from animation. They focus on the transient information effect (cf. Jiang and Sweller, 2022). According to this effect, learning from animation may be hindered if learners are shown too much information in too short a time (e.g., Castro-Alonso et al., 2018). That is, the learners are unable to process the continuous flow of information in working memory. Castro-Alonso et al. (2019) hypothesize that learners with high visuospatial ability should learn more successful from exceedingly transient animations than learners with low visuospatial ability. They summarize various empirical studies that support this hypothesis. However, almost all of these studies focus on the role of spatial ability as assessed by the Mental Rotation Test (e.g., Vandenberg and Kuse, 1978) or the Paper Folding Test (e.g., Ekstrom et al., 1976) and not on visuospatial working memory (for a review see Höffler, 2010).

To identify empirical research that has specifically focused on visuospatial working memory in animation learning, a database search was conducted.

### 2.2 Empirical research

The search was conducted in August 2023 in the Academic Search Premier, APA PsychArticles, APA PsychInfo, and ERIC databases. The search expression was learning AND (animation OR “dynamic visualization” or “dynamic visualisation”) AND (“visual memory” OR “visual working memory” OR “visual short-term memory” OR “visuospatial memory” OR “visuospatial working memory” OR “visuospatial short-term memory”). Further limitations were year of publication (2000–2023), population (human), methodology (empirical study), and language (English).

After removing duplicates, the search yielded 329 articles. Eight additional articles were identified by citations in articles. The following criteria were used to select relevant studies:

- The animation had to be representational, i.e., it had to represent the subject matter to be learned (cf. Ploetzner and Lowe, 2012). Studies that used artistic, decorative, entertaining, or mnemonic animations were not included.
- The animation had to be the main learning material and not merely a minor part of an extensive multimedia environment.
- The visuospatial working memory and the learning performance of the learners had to be assessed.
- The assessed visuospatial working memory and learning performance had to be related.

The titles and abstracts of the 329 articles were read. Occasionally, the full text of an article was skimmed. If an article violated at least one of the criteria, it was excluded. This left 16 articles for full text assessment. The texts of these 16 articles and the eight articles identified by citations were evaluated in their entirety.

Seventeen articles acknowledged the role of visuospatial working memory in learning from animation. However, they did not assess visuospatial working memory (e.g., Moreno and Mayer, 2000, 2002; Yue et al., 2013). In three articles, visuospatial working memory was assessed but not related to learning performance. For example, Boucheix and Guignard (2005) assessed learners’ visuospatial working memory using tasks proposed by Shah and Miyake (1996). Although the learners’ scores on these tasks were used to test whether the experimental groups differed on this variable, they were not related to learning performance. Only four articles met the selection criteria.

Münzer and Stahl (2011) investigated wayfinding learning from static and dynamic visualizations. They assumed that visuospatial representations constructed during learning must be maintained in working memory to successfully perform wayfinding tasks in real buildings. Therefore, they hypothesized that visuospatial working memory capacity—as measured by a Mental Pathway Span Task, a variant of a task originally proposed by Brooks (1967)—would predict wayfinding performance. Contrary to their expectations, the two variables were not correlated. Furthermore, Münzer and Stahl (2011) did not relate their hypothesis to the visualizations used. Thus, their research does not shed light on the role of visuospatial working memory in learning from animation.

Wong et al. (2018) investigated how different variables influence the learning of construction procedures from animation. One of the variables investigated was the learners’ spatial ability. As a component of spatial ability, the capacity of visuospatial working memory was assessed using the Corsi Block Tapping Test (Corsi, 1972). Wong et al. (2018) assumed that spatial ability helps learners process large amounts of rapidly changing information in animated displays. Accordingly, they observed significant small to medium correlations between visuospatial working memory capacity and three performance measures.

In two experiments, Wright et al. (1999) investigated the learning of history from text and from static and dynamic visualizations. They hypothesized that learning from animations would lead to improved learning performance compared to learning from static pictures, and that this improvement would be more pronounced for learners with greater visuospatial working memory. Visuospatial working memory was assessed with a self-constructed Picture Memory Test. In the first experiment, visuospatial working memory correlated significantly with a delayed performance measure in the group who learned from animation. In the second experiment, visuospatial working memory correlated significantly with the same performance measure in two groups who learned from animation. Although the authors summarize that learners who performed well on the visual memory test benefited most from the animations, it remains unclear how visuospatial memory specifically contributed to learning from animation.

More recently, Kühl et al. (2022) investigated how spatial ability influences learning from static and dynamic visualizations. They focused on visuospatial working memory as an important factor of spatial ability. Like Münzer and Stahl (2011), they measured visuospatial working memory using a Mental Pathway Span Task, a variant of a task originally proposed by Brooks (1967). Kühl et al. (2022) found significant (non)linear relationships between visuospatial working memory capacity and learning performance.

Thus, although the importance of visuospatial working memory is often acknowledged in research on learning from animation, empirical investigation of its role in learning is the rare exception. Only four of the 337 articles identified were relevant in this respect. Overall, the results observed in these articles suggest a positive relationship between visuospatial working memory and the performance in learning from animation. However, the variance within the reported results is considerable. Furthermore, only Kühl et al. (2022) and Wong et al. (2018) describe how visuospatial ability may specifically support learning from animation. Thus, there appears to be little systematic empirical research on the role of visuospatial working memory in learning from animation.

## 3 Method and materials

### 3.1 Design and hypotheses

To gain insight into the role of visuospatial working memory, especially in the initial phase of learning from animation, learners were shown two animations of mechanical gears, one of low and one of high complexity, in a within-subject design. The learners had to grasp and memorize the motion patterns of the individual components of the animated gears, i.e., they had to identify event units in terms of the APM. In addition, the learners’ visual working memory (VWM) capacity, spatial working memory (SWM) capacity, and subjective task load were assessed. Four hypotheses were tested:

H1: Since a low complexity animation should be easier to grasp and memorize than a high complexity animation, it is predicted that the low complexity animation will lead to higher learning performance than the high complexity animation.

H2: Due to the difference in complexity of the animations, it is predicted that the subjective task load will be lower for the low complexity animation than for the high complexity animation.

H3: Because learners had to learn the motion patterns of the animations—not their spatial arrangements—the correlation between learning performance and VWM capacity is predicted to be higher than the correlation between learning performance and SWM capacity. This relationship should be more pronounced with respect to the high complexity animation than with respect to the low complexity animation.

H4: As observed in previous studies on subjective task load and learning performance (e.g., Krieglstein et al., 2022), subjective task load and learning performance are predicted to be negatively correlated. Similarly, negative correlations between subjective task load and VWM/SWM capacity are predicted, as lower VWM/SWM capacity should be associated with the perception of higher mental load, at least in the context of complex visual learning tasks.

### 3.2 Participants

With two dependent variables (learning performance on the high and low complexity animation), three predictors (VWM capacity, SWM capacity, and task load), and an expected medium effect size, a power analysis using G*Power (Faul et al., 2007) requires 77 participants to achieve 80% power. A total of 86 students (63 undergraduates and 23 graduates, 70 female and 16 male, mean age *M* = 21.64 years, SD = 2.73) from various study programs at a university in Germany voluntarily participated in the study. They received financial compensation for their participation.

### 3.3 Materials

#### 3.3.1 Animations

**Figure 1:**
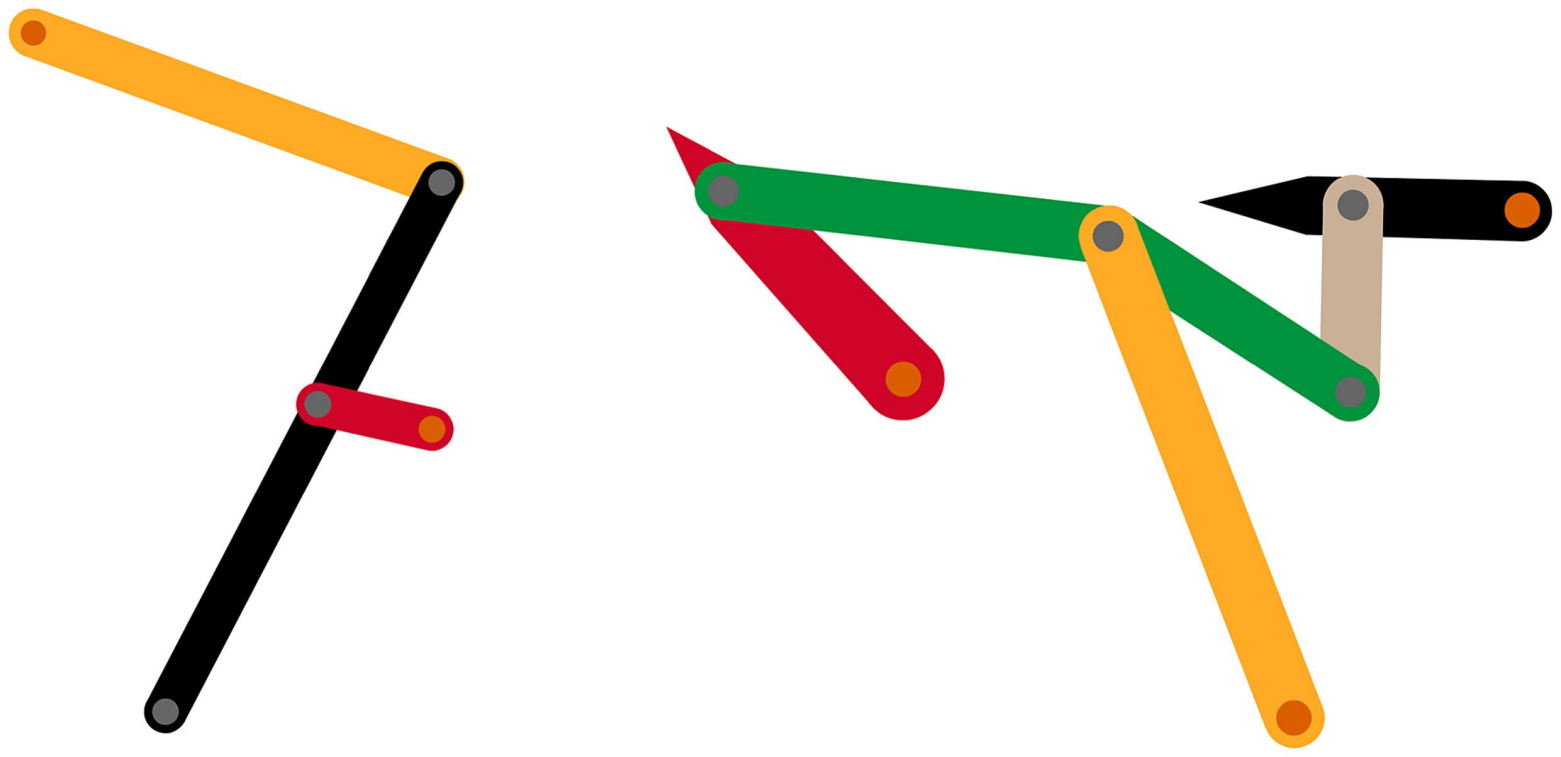
Snapshots of the animations of the four-bar (left) and six-bar (right) linkage.

Two gear mechanisms of varying complexity were animated, a four-bar linkage and a six-bar linkage (see Figure 1). These mechanisms move in one plane and transform the continuous rotation of the input gear (red links) into more complex motion patterns of the output gear (black links). Whereas the four-bar linkage consists of four links—including the rack—and five joints, the six-bar linkage consists of six links—including the rack—and seven joints. Furthermore, while the links of the four-bar linkage have a continuous and regular motion, the links of the six-bar linkage—except for the input gear—have a discontinuous and/or irregular motion. Thus, the two mechanisms differ in both structural and behavioral complexity. The animations were created using Adobe Animate CC.

#### 3.3.2 Assessment of learning performance

**Figure 2:**
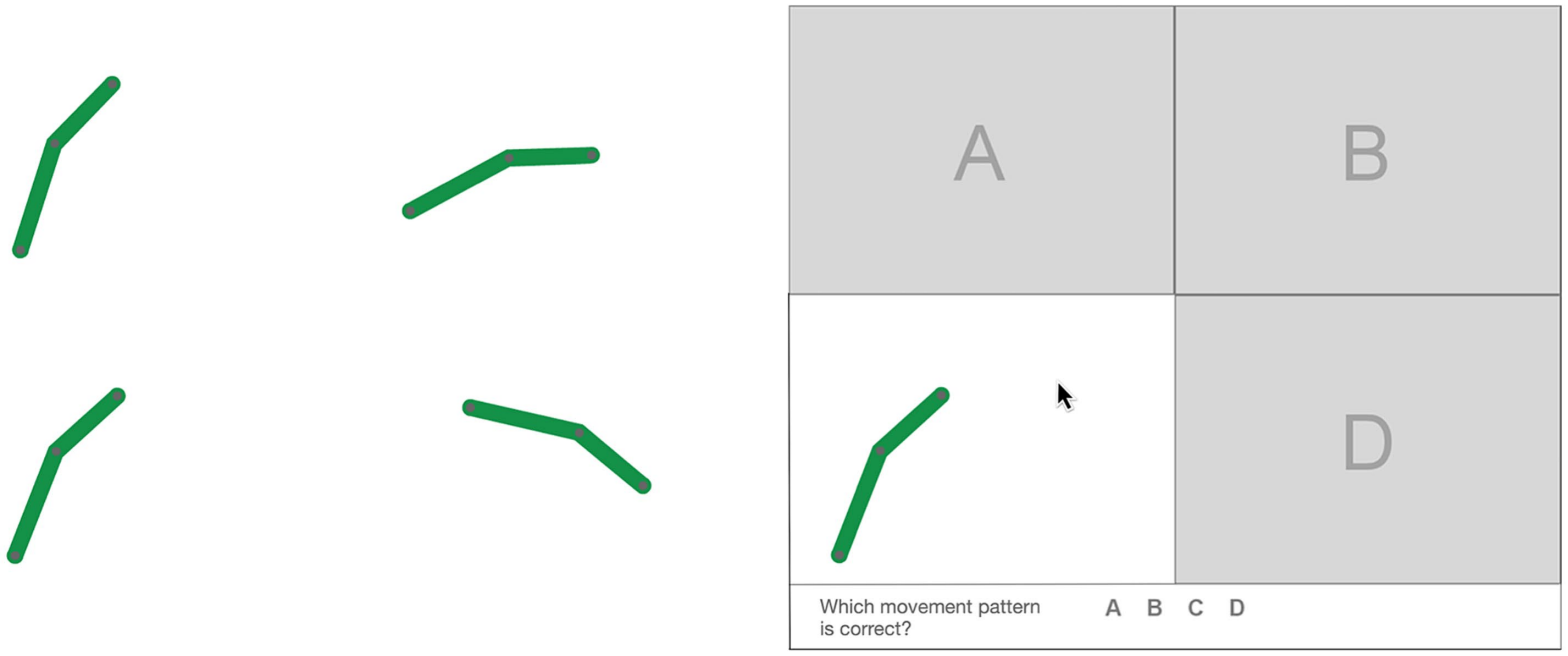
Snapshots of the uncovered (left) and covered (right) correct and incorrect animations of a link.

After learning, the participants had to identify the correct motion pattern of each link. For each link, learners were presented with four animations: the correctly animated version of the link and three incorrectly animated versions of the link. The incorrect versions moved in the same direction and traveled the same path in the same time as the correct version of the link, but had different motion patterns along the path (e.g., continuous vs. discontinuous motion). Figure 2 shows snapshots of the four animations of the green link of the six-bar linkage at 3 s. As the animations have different motion patterns, they are only in the same positions and have the same orientations at the beginning and at the end of a loop. To avoid confusing the learner with four animations running simultaneously, the animations were covered independently. When the mouse was moved over a cover, the looping animation became visible (see Figure 2). This allowed learners to watch one animation after another and move the mouse back and forth between the animations for as long as they wanted. The animations of the different links were shown to the learners in random order. The learners did not receive any feedback on their learning performance. All animations were created using Adobe Animate CC.

#### 3.3.3 Visual Patterns Test and Corsi Block Tapping Test

To assess the visual component of the learners’ visuospatial working memory, a computerized version of the Visual Patterns Test (Della Sala et al., 1997) was used. Participants are shown increasingly complex matrix patterns consisting of 50% black and 50% white squares. The matrices range from 2 × 2 matrices to 5 × 6 matrices. Each matrix of a certain complexity is shown with three different patterns. A pattern is presented to the learner for 3 s. The learners are then asked to reproduce the pattern in an empty matrix. If all three patterns of a given complexity cannot be reproduced, the test is stopped. The complexity of the last pattern that was successfully reproduced determines the visual memory span. When the three 2 × 2 matrices are used for practice, the span ranges from 3 to 15. As this study is not intended for clinical diagnosis, the more sensitive score of all correctly reproduced patterns is used (cf. Della Sala et al., 1997). A maximum of 39 points could be achieved. Learners were given feedback on their performance.

To assess the spatial component of the learners’ visuospatial working memory, a computerized version of the Corsi Block Tapping Test (Corsi, 1972) was used. In the computerized version, nine identical and spatially separated squares are placed within a larger rectangle. Learners are presented with increasingly long sequences of squares, which are highlighted one at a time. Highlighting a square and moving from one square to another takes 1 s each. The learner is asked to reproduce a sequence by clicking on the corresponding squares in the correct order. Sequences range from two squares up to nine squares. Two different sequences are shown for each length. The spatial layout of the squares and the sequences of highlighted squares were arranged according to the standardized administration of the Corsi Block Tapping Test as proposed by Kessels et al. (2000). If both sequences of a given length cannot be reproduced, the test is stopped. The length of the last sequence successfully reproduced determines the block span. When sequences of length two are used for practice, the span ranges from 3 to 9. As this study is not intended for clinical diagnosis, the more sensitive score of all correctly reproduced sequences is used (cf. Kessels et al., 2000). A maximum of 14 points can be achieved. Learners were given feedback on their performance.

#### 3.3.4 NASA Task Load Index

Five scales of a computerized version of the NASA Task Load Index (TLX, Hart and Staveland, 1988) were used to assess learners’ perceived task load: mental demand, temporal demand, overall performance, effort and frustration. The physical demands scale was not appropriate for this study. Each scale was rated by the learners on a 20-point Likert scale. As suggested in the TLX manual, the scales were weighted according to their supposed importance to the learning task. The following weights were applied: mental demand 5, effort 4, temporal demand 3, performance 2, and frustration 1. The TLX score is the weighted sum of the scores on each scale. A maximum task load of 300 can be achieved.

### 3.4 Procedure

The students were investigated in groups of up to eight individuals. Each student learned individually using a computer equipped with a 21-in. screen and a mouse. The computers were placed on separate desks. All the material was presented to the students using a computer program created with the authoring software ActivePresenter.^1^ The size of the presentation area was 1,200 × 1,000 pixels.

First, the students were told how to use the program and what they would be learning. The students were then asked to give their consent to take part in the study. The program then presented the material to the students in the following order: (1) example of an oscillating link and identification of its motion pattern, (2) first gear and identification of its motion patterns, (3) first TLX assessment, (4) second gear and identification of its motion patterns, (5) second TLX assessment, (6) Visual Patterns Test, and (7) Corsi Block Tapping Test. Half of the students received the lower complexity animation first and then the higher complexity animation. The other participants received the animations in reverse order. The procedure took approximately 40 min.

## 4 Results

Regarding the first hypothesis, learning performance was significantly higher for the low complexity animation (*M* = 76.74%, *SD* = 28.50) than for the high complexity animation (*M* = 61.86, *SD* = 21.17; *t*(85) = 4.36, *p* < 0.001). According to Cohen (1988), the effect size is medium (*g* = 0.46).

Correspondingly, and regarding the second hypothesis, subjective task load was significantly lower for the low complexity animation (*M* = 159.44, *SD* = 35.40) compared to the high complexity animation (*M* = 203.42, *SD* = 34.76; *t*(85) = 10.74, *p* < 0.001). According to Cohen (1988), the effect size is large (*g* = 0.88).

Consistent with the third hypothesis, Table 1 shows that the correlation between learning performance and VWM capacity is higher than the correlation between learning performance and SWM capacity. As predicted, this relationship is more pronounced with respect to the high complexity animation than with respect to the low complexity animation.

**Table 1:** Intercorrelations between learning performance, task load (TLX), visual (VPT), and spatial (Corsi) working memory capacity.

Variable	Perf. low compl. anim.	Perf. high compl. anim.	TLX low compl. anim.	TLX high compl. anim.	VPT	Corsi
Perf. low compl. anim.	—					
Perf. high compl. anim.	0.22^*^	—				
TLX low compl. anim.	−0.35^**^	−0.15	—			
TLX high compl. anim.	−0.23^*^	−0.24^*^	0.41^**^	—		
VPT	0.16	0.52^**^	−0.18	−0.25^*^	—	
Corsi	0.10	0.10	0.07	0.07	0.40^**^	—

* The correlation is significant at the level of *α* = 0.05.

** The correlation is significant at the level of *α* = 0.01.

In line with the fourth hypothesis, there are significant negative correlations between subjective task load and learning performance for both animations (see Table 1). Furthermore, as predicted, subjective task load and VWM capacity are negatively correlated. Subjective task load and SWM capacity are almost uncorrelated.

To further analyze the strength of the relationships examined, two hierarchical regression analyses were conducted. In each analysis, the dependent variable was learning performance, and the predictors were subjective task load, VWM capacity, and SWM capacity. The criterion for including a predictor was that its increment in explaining the variance of the dependent variable was significant at the level of *α* = 0.05. For the low complexity animation, only subjective task load was a significant predictor of learning performance (*β* =−0.35, *F*(1, 84) = 11.43, *p* < 0.01). According to Cohen (1988), the effect size is medium. For the high complexity animation, only VWM capacity was a significant predictor of learning performance (*β* = 0.52, *F*(1, 84) = 31.28, *p* < 0.001). According to Cohen (1988), the effect size is large.

## 5 Discussion

A systematic database search revealed that the role of visuospatial working memory has rarely been investigated in research on learning from animation. This is surprising given that visuospatial working memory plays a central role in the processing of visuospatial information (e.g., Luck and Hollingworth, 2008; Schurgin, 2018) and that current theories describe learning from animation as, among other things, a visual and spatial learning task (Lowe et al., 2022). Instead, animation research often relies on subjective measures of task load to explain and predict learning performance (e.g., Hasler et al., 2007; Moreno, 2007; Wong et al., 2012).

To investigate the relationship between visuospatial working memory and animation processing, a correlational study was conducted. In the study, learners were required to complete visual learning tasks involving two animations of varying complexity. Learners’ VWM capacity, SWM capacity, and perceived task load were assessed. This made it possible to formulate differential hypotheses about how VWM, SWM, and task load are related to the processing of animations of low and high complexity, respectively.

As predicted, the results are different for each animation used. For the low complexity animation, VWM and SWM capacity correlate very little with learning performance. This may indicate that the learners’ perceptual resources did not seriously limit their achievement of this learning task. Nevertheless, learners had to direct their visual attention to the displayed links and identify and memorize their motion patterns. The effort involved in these processes is probably reflected in the moderate negative correlation between perceived task load and learning performance. As VWM and SWM capacity were not limiting factors in this learning task, only perceived task load significantly predicted learning performance.

For the high complexity animation, the picture is very different. Learners not only had to learn movements of more links, but also more complicated movements involving irregular and non-continuous motion. The large correlation between VWM capacity and learning performance may indicate that VWM was a limiting factor in achieving this learning task. As with the low complexity animation, SWM capacity correlates little with learning performance. Although perceived task load increased significantly from the low to the high complexity animation, the correlation between task load and learning performance did not. In this learning task, only VWM capacity significantly predicted learning performance.

The finding of moderate to high correlations between VWM capacity and learning performance on the one hand, and only weak correlations between SWM capacity and learning performance on the other, emphasizes that the learning tasks were visual rather than spatial. While this design of the learning tasks was intentional, the current study does not shed light on the question of how VWM and SWM capacity relate to animation learning when the learning tasks become more spatial. Future research could also enhance the findings of the current study by experimentally investigating the influence of visuospatial working memory on animation learning, for example by examining samples paired by their visuospatial working memory capacity.

Overall, the results support current theories of learning from animation such as the APM in which it is assumed that visuospatial working memory affects the early stages of animation processing, where bottom-up perceptual processes are prominent. However, the current study leaves open the question of how visuospatial working memory relates to later phases of animation processing as described in the APM. Theoretically, stronger relationships would be expected in the early phases of animation processing and weaker relationships in the later phases when cognitive processes come to the fore.

A better understanding of the role of visuospatial working memory in learning from animation may not only help to further validate theoretical models of animation learning, such as the APM, but also to support learning by tailoring the design and use of animation to learners’ perceptual and cognitive resources.

## Data Availability

The raw data supporting the conclusions of this article are available from the Open Science Foundation, https://osf.io/yqprf/.

## Footnotes

^1^ atomisystems.com
